# Use of geographic indicators of healthcare, environment and socioeconomic factors to characterize environmental health disparities

**DOI:** 10.1186/s12940-016-0163-7

**Published:** 2016-07-22

**Authors:** Cindy M. Padilla, Wahida Kihal-Talantikit, Sandra Perez, Severine Deguen

**Affiliations:** Department of Quantitative Methods in Public Health, EHESP School of Public Health, Sorbonne-Paris Cité, 35043 Rennes, France; Department of Environmental and Occupational Health, EHESP School of Public Health, Sorbonne-Paris Cité, 35043 Rennes, France; INSERM U1085-IRSET – Research institute of environmental and occupational health, Rennes, France; UMR ESPACE 7300, University of Nice Sophia, Nice, France

**Keywords:** GIS, Healthcare accessibility, Environment, Infant mortality, Environmental health inequalities

## Abstract

**Background:**

An environmental health inequality is a major public health concern in Europe. However just few studies take into account a large set of characteristics to analyze this problematic. The aim of this study was to identify and describe how socioeconomic, health accessibility and exposure factors accumulate and interact in small areas in a French urban context, to assess environmental health inequalities related to infant and neonatal mortality.

**Methods:**

Environmental indicators on deprivation index, proximity to high-traffic roads, green space, and healthcare accessibility were created using the Geographical Information System. Cases were collected from death certificates in the city hall of each municipality in the Nice metropolitan area. Using the parental addresses, cases were geocoded to their census block of residence. A classification using a Multiple Component Analysis following by a Hierarchical Clustering allow us to characterize the census blocks in terms of level of socioeconomic, environmental and accessibility to healthcare, which are very diverse definition by nature. Relation between infant and neonatal mortality rate and the three environmental patterns which categorize the census blocks after the classification was performed using a standard Poisson regression model for count data after checking the assumption of dispersion.

**Results:**

Based on geographic indicators, three environmental patterns were identified. We found environmental inequalities and social health inequalities in Nice metropolitan area. Moreover these inequalities are counterbalance by the close proximity of deprived census blocks to healthcare facilities related to mother and newborn. So therefore we demonstrate no environmental health inequalities related to infant and neonatal mortality.

**Conclusion:**

Examination of patterns of social, environmental and in relation with healthcare access is useful to identify census blocks with needs and their effects on health. Similar analyzes could be implemented and considered in other cities or related to other birth outcomes.

## Background

Geographical inequalities have become a major issue which guides policy development in Europe. The inhomogeneity of the environment on the territory, does not guarantee an equal access to an environment of quality [1]. In the same way, unequal distribution of people's exposure to – and potentially of disease resulting from – environmental conditions constitutes an important public health concern in Europe [2].

Reducing health inequalities involves the characterization and the identification of how factors accumulate and interact in an area. Certain socioeconomic groups bear a disproportionate burden of environmental externalities [3], and vulnerable to the health effects resulting from this exposition [4]. Previous studies have demonstrated that population with a low socioeconomic status tends to be more highly exposed to air pollutants and toxicants, due especially to their residential proximity to pollution sources (e.g. high-traffic roads, industrial facilities and waste disposal sites) [5–12]. Conversely, few have considered to wholesome environments may be related to urban socioeconomic inequalities [13] and have shown that access to green spaces may have a beneficial effect on health [14, 15].

Infant and neonatal mortality are highly sensitive measures which reflect economic development, general living conditions, social well-being and rates of illness of whole populations [16]. Moreover, they are recognized by the World Health Organization (WHO) as indicators of the health status of a population and of the effectiveness of the health care system [16]. Skilled assistance at delivery and access to emergency obstetric care are the most effective interventions to prevent these early and intra-partum related deaths [17]. This requires both the availability of such services as well as the will and the possibility for pregnant women to seek this care at delivery [18]. Recent research has considered that accessibility to health care facilities is known to influence health services usage [19] but the quality of life depends on the adequacy of their position in the territory [19, 20]. Most of these neonatal deaths occur during the first day of life and complications related to delivery care make up a large proportion of the overall neonatal mortality [21, 22]. Contextual factors, such as social and environmental exposures, are well-documented to be associated with adverse birth outcomes. Children's exposure to air pollution is therefore of primary concern [23], especially in reference to the life course approach which state that health problems during childhood have repercussions on health at later stages of life [24]. They are particularly sensitive to environmental factors such as teratogens agents, and early exposure to environmental factors can lead to diseases or subsequent severe functional deficits [25–27].

Previous studies demonstrated that the adequate location of healthcare facilities deserve careful and detailed future analysis. Geographical factors such as distance between home and healthcare facilities are part of the first and the second delay and suggested an influence on the choice of delivery place [18] as well as being related to neonatal mortality risks [28, 29]. Some studies demonstrated that the usage of health services decreases with increasing distance between health facilities and families’ homes. This also holds true in the case of accidental out-of-hospital births [30, 31]. In France, distance to the closest maternity unit was found to aggravate the risks of out-of-hospital birth [30].

Several factors hinder access to the health care services needed to avert maternal and newborn deaths and morbidity. These include perception of the mothers as cultural norms, attention to the needs of women in planning and delivering health services, previous experiences in the hospital [32], and individual factors or health behaviors of the mother as maternal age (teenage mothers and mothers aged 40 and over), multiple births, smoking habits, obesity, that could affect health outcomes [33].

Moreover, the health status of a given population is a result of complex interactions between the social and physical characteristics of their areas of residence (e.g. neighborhood socioeconomic status), environmental exposure factors and those that relate to the characteristics of the health care system and care practices. To our knowledge, no previous studies explored such a large variety of field to characterize neighborhood areas.

At the small area level, we view to identify the census blocks which cumulate environmental inequalities where public authorities should design relevant preventive actions in priority. In our context, we aim to identify through geographic indicators of healthcare, environment and socioeconomic factors, local territorial inequalities. The two majors objectives were : i) Identification and characterization of socioeconomic, health accessibility and exposure factors in order to explain how they accumulate and interact in an area. ii) Investigation the role of these three fields on environmental health inequalities related to infant and neonatal mortality.

## Methods

### Study setting and geographical unit

This ecological study setting in the Nice metropolitan area (MA), situated in Provence Alpes Côte d’Azur region, southern France. The geographical unit is the sub-municipal French census block called IRIS (Îlot Regroupé pour l'Information Statistique) defined by the National Institute of Statistics and Economic Studies (INSEE). It is the smallest administrative unit for which socioeconomic and demographic data are available in France. This geographical unit averages 2000 inhabitants and is constructed to be as homogenous as possible in terms of socio-demographic characteristics and land use. Nice MA has an approximate population of 537 769 habitants in 2012 divided into 49 municipalities and 236 census blocks, for a total area of 1 465,8 km^2^. Nice MA is composed by a majority of women compare to men; the specificity of the MA is that families with or without children leaved the Coast to the periphery, in search of a bigger housing environment with lesser cost. On the contrary, single-parent families or only people leave this space consisted mainly of individual housing to get closer to the Coast. Moreover, Nice MA is characterized by a heterogeneous distribution of the environment due to the geographic repartition of the MA. Whereas Nice is characterized by a dense urban area with an important traffic related urban pollution close to the littoral coast, conversely, the mountain part is sparse and the density of population is very low.

### Geographical indicators

The construction of geographic indicators is needed to identify and characterize at the census block level the level of; deprivation, environmental exposure and accessibility measure to healthcare facilities. They were calculated using the Geographical Information System (GIS) with ArcGIS version 9.1 (ESRI, Redlands, CA).

### Deprivation index

This index used to characterize the socioeconomic dimensions have been defined and analyzed in previous studies in order to analyze environmental and health inequalities [12, 34–36].

The deprivation index built at the French census block level was developed by Lalloue et al in 2013 [37]. The index used multiple principal component analysis. It is constructed from 13 variables which come from the National Institute of Statistics and Economic Studies available variables. It is a cumulative index that combines different dimensions family structure, household type, immigration status, mobility, employment, income, education and housing.

### Environmental exposure “negative and positive”

**Fig. 1:**
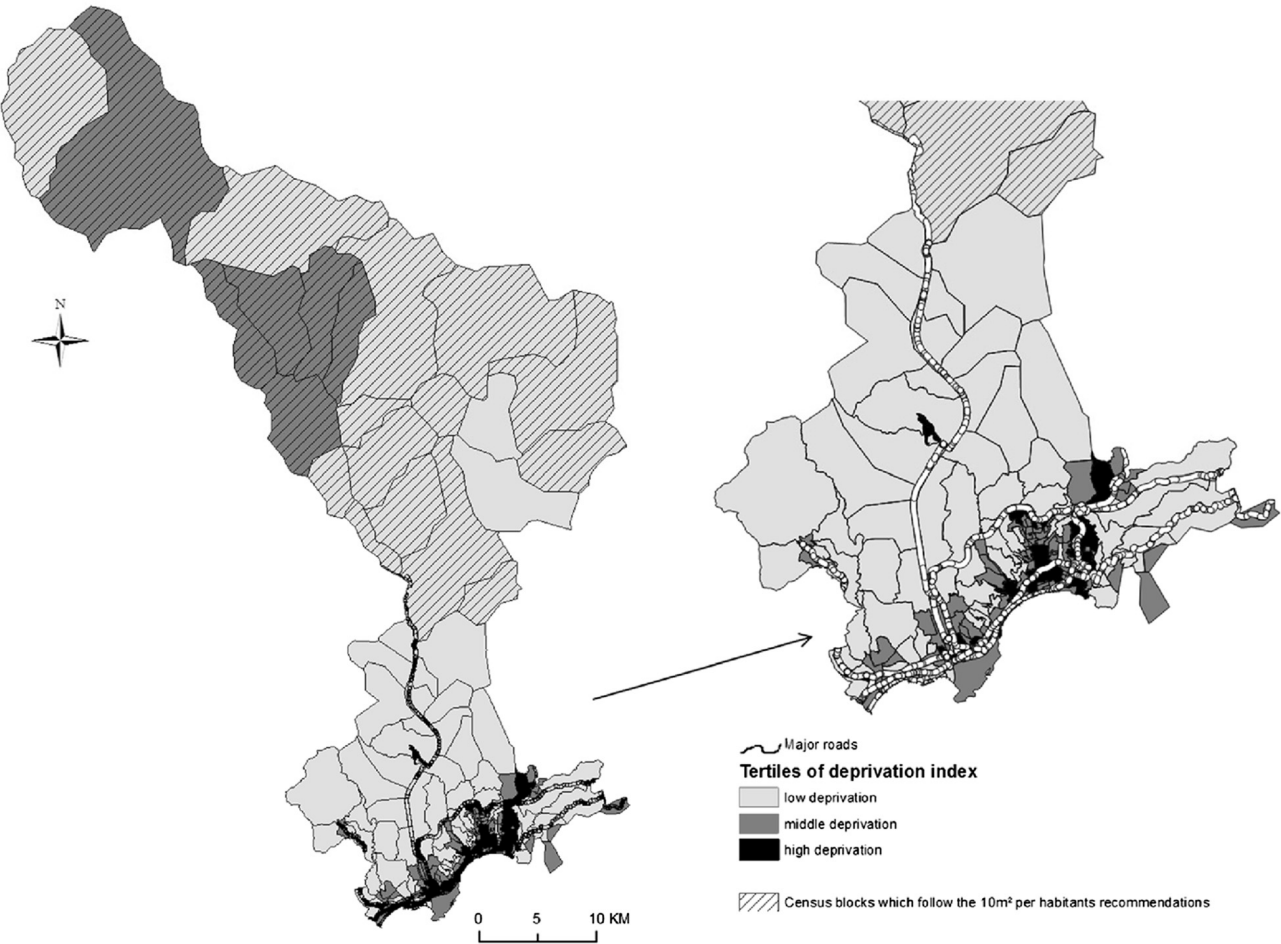
Spatial distribution of the environmental inequalities factors: major streets, census blocks who follow the 10 m^2^ of green space per habitants recommendations, deprivation index by tertiles

The high-traffic roads database and topographic database information was provided from the National Geographic Institute. Proximity to major roads with high traffic was determined as the proportion of residential building located within a specified distance of the high-traffic roads (minimum 70 km/h) in each census block [38]. More precisely, circles with radii of 150 m were created around each road segment according to the literature [38, 39]. The measure of proximity was determined by select residential buildings located inside each circle which will be considered exposed to the high-traffic road and the measure for census block was obtained by dividing the total number of exposed residential building by the total building of the census block. Figure 1 presents the spatial distribution of the environmental exposure factors: major streets, census blocks who follow the 10 m^2^ of green space per habitant recommendations and the deprivation index by tertiles according to its distribution.

### Proximity to green space

The green space databases were providing by the Corine Land Cover website [40]. The definition of green space included natural area (e.g. parks, forest, garden…); to not include pesticide in our green space definition, agricultural areas have not been taken into account. Two green space indicators have been created. The first indicator is defined by the proportion of the geographic area occupied by green spaces within the total area of a census block [41, 42]. The second indicator is defined according to the World Health Organization (WHO) guidelines which recommends at least 10 m^2^ of green space per habitant [43, 44].

### Accessibility to healthcare

The present work focuses on the spatial dimensions accessibility and availability to healthcare service. Spatial accessibility refers to the distance to the nearest healthcare service whereas spatial availability is defined as the amount of healthcare services available in a predefined area. First, the proximity indicator was defined as the road network distance (measured in meters and in minutes) to the closest healthcare service related to the mother (gynecologist, midwives and hospital center with a specialized service for pregnant women) and the same indicator related to newborn (pediatricians and hospital center with a specialized service for newborn). Second, the availability indicator is defined as the medical density which represents the number of healthcare service within the female population of reproductive age of the census block.

### Quantification of mortality impacts

**Fig. 2:**
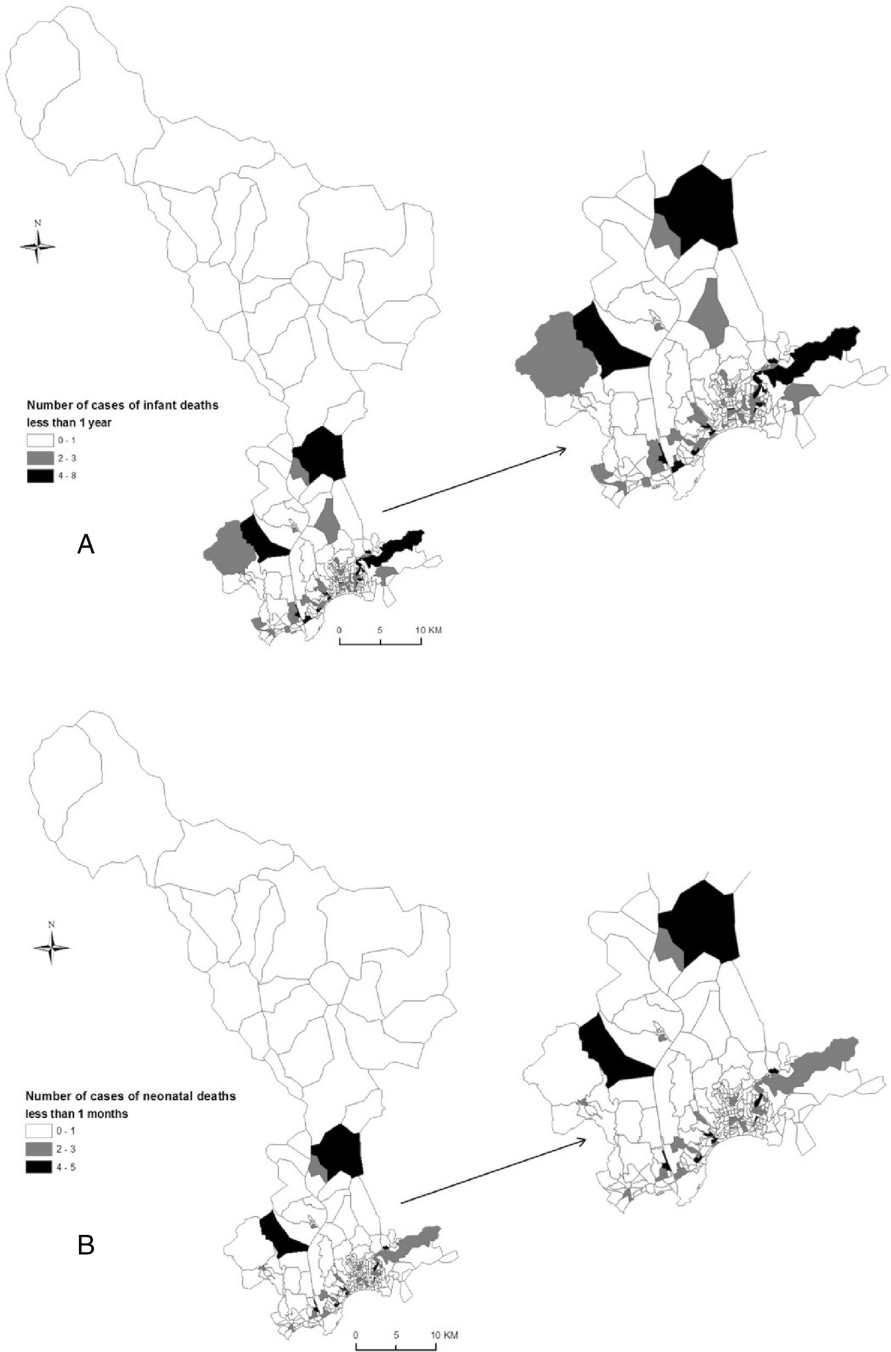
Spatial distribution of number of cases of infant (**a**) and neonatal (**b**) deaths by tertiles at the census blocks level of Nice MA

Neonatal and infant mortality are defined as the number of babies who died during their first month and their first year of life per 1000 live births that occurred during this time period, respectively. Cases were collected from death certificates in the city hall of each municipality in the Nice MA. Using the parental postal addresses, cases were geocoded to their census block of residence using ArcGIS version 9.1 (ESRI, Redlands, CA). The French data protection authority (CNIL) approved the study (number: 911149v1). Figure 2 illustrates the spatial distribution of number of cases of infant and neonatal deaths by tertiles at the census blocks level of Nice MA.

### Statistical analysis

In our ecological study, to be robust in the following statistical analysis, one of the main features is to measure the degree of interdependence between located observations. We use basic statistical analysis after checking that spatial autocorrelation is no statistically significant (data not shown). Analyses of correlations between geographic indicators were performed using Spearman’s Correlation test after proving the non-normal distribution of these indicators.

Firstly, we create a **classification** using a Multiple Component Analysis (MCA) following by a hierarchical clustering. This methodology allow us to characterize the census blocks in terms of environmental patterns according to level of socioeconomic, environmental and accessibility to healthcare, which are very diverse definition by nature. MCA analysis is one of the most popular dimensionality reduction methods dealing with categorical variables. The MCA method permits to take variable collinearity into account, thereby avoiding redundant information. Hierarchical clustering is frequently used after MCA [45] in data mining to create meaningful categories. A distance between elements (usually the Euclidian distance) and a distance between categories are defined. Thanks to the Ward’s distance, this algorithm allows to obtain homogeneous categories in their composition and heterogeneous between them (i.e. with maximum between-categories inertia). These analyses were made with FactoMineR, a R package dedicated to multivariate Exploratory Data Analysis [46].

Secondly, we performed a standard **Poisson regression model** for count data after checking the assumption of overdispersion. We examined the relation between infant and neonatal mortality rate and the three environmental patterns which categorize the census blocks after the classification. All analyses were conducted using STATA, Statistical Software: Release 13. College Station, TX: StataCorp LP.

## Results

**Fig. 3:**
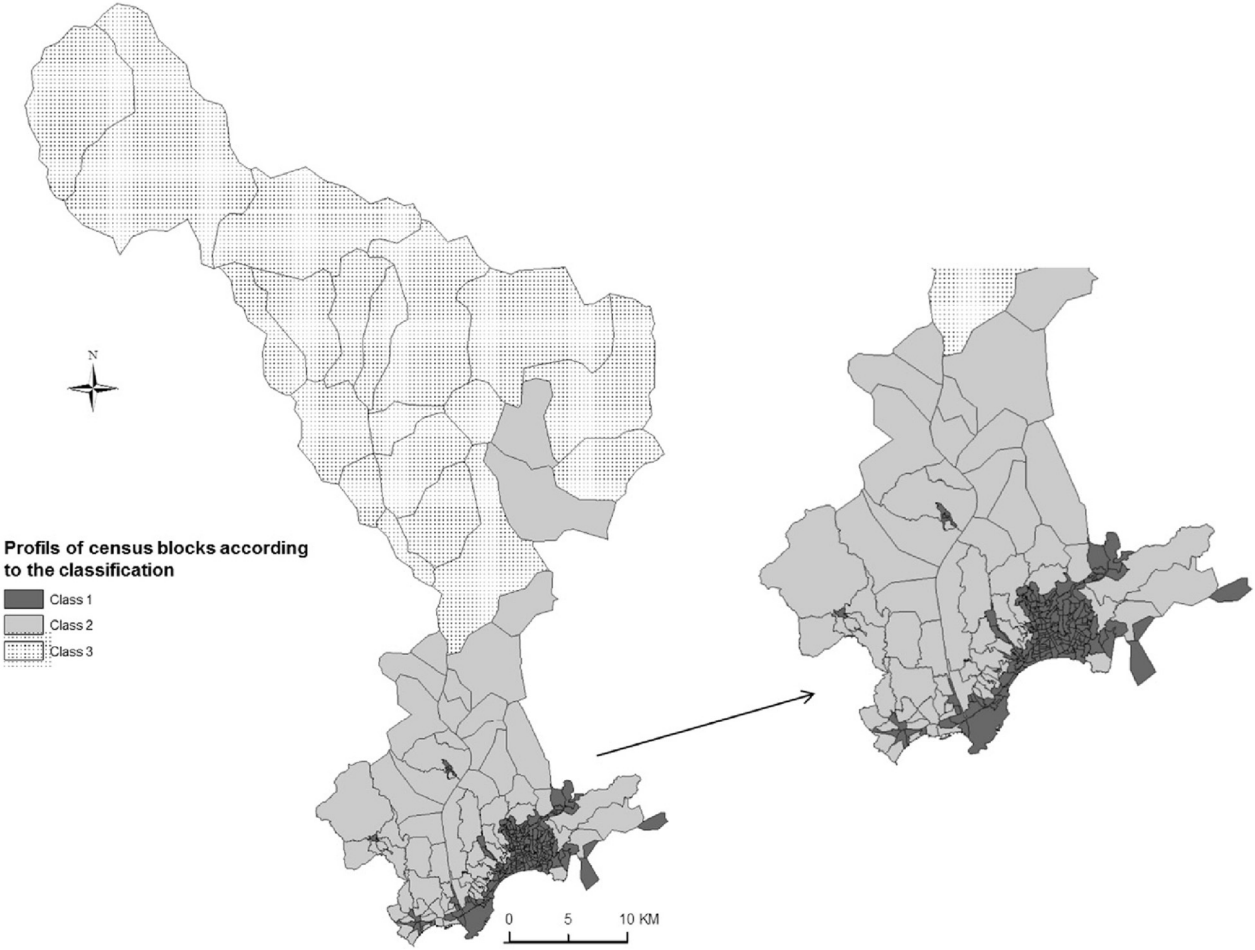
Spatial distribution of the census blocks profiles in three environmental patterns according to the social, environmental and accessibility of healthcare classification

We identified three environmental patterns according to environmental exposure, deprivation and accessibility to healthcare. The percentage of variance explained by the two first components is 39 %. Figure 3 present the spatial distribution of the census blocks according to their profiles. The environmental pattern 1 represents census blocks faced environmental inequalities but with favorable accessibility of healthcare and transportation networks. The higher and some middle deprived census blocks faced higher proximity to high-traffic roads and lower proportion to green space related to the census blocks surface and the WHO recommendations. However, these census blocks have higher availability and a very easy accessibility of healthcare and very good transportation networks. The environmental pattern 2 represents census blocks exposed to a mixture of positive and negative factors which balance each other. This class is composed by some higher and all the middle deprived census blocks who lived close to high-traffic roads but close to green space too and in the middle class in term of accessibility of healthcare. The environmental pattern 3 encountered only favored very green census blocks but with an unfavorable accessibility to healthcare. The details characteristics of the categories are presented in Table 1.

**Table 1:** Description of the characteristics of the 3 environmental pattern of census blocks according to their level of environmental exposure, deprivation and accessibility of the nearest healthcare after the classification

		Environmental patterns		
		Pattern 1 *N =* 147	Pattern 2 *N =* 70	Pattern 3 *N =* 19
Environmental exposure	% of green space in a CB †	4.35 ± 11.78^a^	37.67 ± 29.79	84.51 ± 15.81
	% of CB with 10 m^2^ per habitant	0 %	1.43 %	100 %
	% of CB with habitants living less than 150 m to roads	64.63 %	54.29 %	5.26 %
Accessibility of Healthcare †	Density of specialist/1.000 women	1.85 ± 5.92	0.52 ± 2.41	0
	Distance to healthcare for women (meters)	1141 ± 1704	4653 ± 4511	30334 ± 15473
	Distance to healthcare for newborn (meters)	1168 ± 1703	6706 ± 8340	53908 ± 14877
	Time to nearest healthcare for women (minute)	0.94 ± 1.69	4.87 ± 5.48	40.79 ± 16.15
	Time to nearest healthcare for newborn (minute)	2.47 ± 0.62	6.98 ± 9.43	53.18 ± 12.60
Index of deprivation	% Low deprived	6.80 %	80 %	68.42 %
	% Middle deprived	39.46 %	20 %	31.58 %
	% High deprived	53.74 %	0 %	0 %
Mobility	% population who work in the city of residence	71.41 ± 15.35	41.38 ± 21.28	39.83 ± 23.50
	% population who travel by public transport	23.86 ± 8.74	6.59 ± 2.74	4.13 ± 2.79
	% population who travel by foot	16.05 ± 9.54	4.55 ± 3.33	14.69 ± 12.79
	% population who travel by bike	10.01 ± 3.84	7.24 ± 3.75	3.49 ± 7.09
	% population who travel by car	46.09 ± 13.62	78.10 ± 5.99	72.46 ± 15.05

*CB* census blocks

^a^mean ± standard deviation

Table 2 shows the correlation between the geographic indicators: the deprivation index, the proximity to high-traffic roads, the proportion of green space into census blocks and the quantitative measurement of healthcare facilities into census blocks. Deprivation index is significantly and positively correlated to the proximity to high-traffic roads within 150 m and it is significantly but negatively correlated to the proportion of green space and Distance/Time to healthcare facilities related to mother and newborn. Proximity to high-traffic roads indicator is significantly but negatively correlated to the proportion of green space and it is significantly but positively correlated to the distance/time to healthcare facilities related to mother and newborn. As represented in Fig. 1, the most deprived neighborhood are located in the urban area of the city of Nice on the coastline, while the most favored neighborhoods are located outside the urban areas in the mountain part of the metropolitan area.

**Table 2:** Spearman’s Correlation Matrix of geographic indicators at the census blocks (CB) level

Variables	Deprivation index	Proximity to roads 150 m	Proportion of green space into CB	Quantitative measurement of healthcare facilities into CB
Deprivation index	1			
Proximity to roads 150 m	0.39*	1		
Proportion of green space into CB	−0.61*	−0.33*	1	
Quantitaive measurement of healthcare facilities into CB	0.19	0.18	−0.30*	1
Distance/time to nearest heathcare facilities related to newborn	−0.66*	0.48*	0.74*	−0.53*
Distance/time to nearest heathcare facilities related to mother	−0.57*	0.48*	0.77*	−0.45*

*Bonferroni adjusted significant level *p <* 0.01

The infant mortality rate is equal to 3.43 per 1000 live births and the neonatal mortality rate is equal to 2.41 per 1000 live births for Nice MA between 2000 and 2012. The relative risks of infant and neonatal mortality according to the level of deprivation index in the census blocks are equal to RR = 1.14 95%CI [1.02; 1.27] and RR =1.15 95%CI [1.01; 1.32] with respected p values *p =* 0.026 and *p =* 0.041. Table 3 presents the relative risks of infant and neonatal mortality according to the classification of the census blocks taking into account positive and negative environmental exposures, deprivation index and accessibility of healthcare facilities related to mother and newborn. Despite a trend of the infant and neonatal mortality rate between the environmental pattern 1 to the environmental pattern 3, the p value =0.17 for the relative risk of infant mortality in the census blocks of the environmental pattern 2 compare to the environmental pattern 1 RR = 0.83 95%CI [0.64; 1.08]. However, it should be noted that the number of census blocks by environmental pattern are very small so the significance of the results must be interpreted with caution.

**Table 3:** Counts, Rate and Relative risk (RR) of Infant and neonatal mortality according to the classification of the census blocks

	Infant Mortality			Neonatal Mortality		
	Number of cases	Rate	RR [95 % CI]	Number of cases	Rate	RR [95 % CI]
Environmental patterns						
Pattern 1 *N =* 147	203	3.55	1	142	2.51	1
Pattern 2 *N =* 70	77	3.32	0.83 [0.64; 1.08]	57	2.38	0.88 [0.65; 1.20]
Pattern 3 *N =* 19	4	3.01	1.17 [0.43; 3.14]	2	1.78	0.83 [0.21; 3.37]

rate : per 1000 live births

*CI* confidential interval

## Discussion

In summary, our investigation conducted at the census blocks level showed that there are environmental inequalities and social inequalities link to infant and neonatal mortality in Nice MA. Moreover, these inequalities are counterbalance by the close proximity of deprived census blocks to healthcare facilities related to mother and newborn. Using GIS for geographic indicators and a combination of MCA and clustering analysis, we identified three environmental patterns which combined multiple factors and how they accumulate in a small area. Despite a significant relation on infant and neonatal mortality risk according to the level of deprivation index, our results did not reveal environmental health inequalities after taking into account the combined effect.

Regarding study strengths, first, the methodology presented here to create classification of the census blocks according to multiple factors could allow us to relatively easily analyze environmental health inequalities. Moreover, similar approaches could be implemented related to; chronic disease which are sensible in the environmental context as cardiovascular, cancer and diabetes disease or could consider other cities. Second, to our knowledge, this is the first study which simultaneously uses a rich set of geographical indicators as positive and negative environmental exposures, deprivation and accessibility to healthcare facilities according to mother and newborn at the census blocks level to take into account the combined effect on environmental health inequalities. This is an important strength because populations are rarely exposed to a single factor at their place of residence.

Our study has some weakness related to missing integrated information in the classification of the census blocks. Firstly, we are not able to consider living conditions such as the indoor physical environment such as water, sanitation and air quality or housing conditions such as crowding, housing accessibility and safety, thermal comfort (i.e. heat and cold), and energy affordability [47, 48]. Housing conditions are one of the mechanisms by which social and environmental inequality translates into health inequality [49]. Secondly, we have no data on the estimated or measured dioxide azote (NO_2)_, particulate matter (PM) or others air pollution exposure which is likely to represent the largest single environmental health risk, due to adverse births outcomes [50–55]. However, an indicator of proximity to high-traffic roads could be a good proxy of air pollution related to traffic [38]. Thirdly, we could not therefore assess the cumulative negative outdoor exposure with proximity to high-traffic roads, noise, and industrial activity or residential heating due to lack of having no data.

In this study, we adjusted for neighborhood deprivation, but there are additional risk factors hypothesized in the literature for which we unfortunately do not have at the individual-level. Incorporating information from maternal interviews or detailed medical records, which we did not have available for this study, could help control for the potential influence of these factors. For example, birth weight, gestational age, mother’s age, and parity of the newborn have been linked with risk of infant mortality. Moreover, some maternal lifestyle behaviors have been linked including the consumption of alcohol, smoking, using drugs, maternal nutritional deficits and access to health care [56–58]. Instead, we considered a deprivation index used in previous articles related on environment health inequalities in France, which is known to be closely related to individual socio economic factors used in studies conducted at fine spatial scale [59].

We know that geographic level is an important consideration in such investigations. The size has to be as small as possible to maximize the homogeneity of specified variables within each area, as well as large differences between areas. In this study we assume the major limitation due to the choice of the geographic level. In fact individual members of a census block all have the average characteristics of the group as whole, when in fact any association observed between variables at the group level does not necessarily mean that the same association exists for an individual plucked from the census block. Due to the ecological design of our study, the results obtained at the census block level cannot be extrapolated to the individual level. Another limitation in our study is the small study population according to the number of the census blocks corresponding on the sample size of the study. We used the smallest spatial level for with socioeconomic variables are routinely available in France.

Investigating associations between the socioeconomic status and the environmental level of exposure have suggested the existence of environmental inequalities. We found that high deprived census blocks were more exposed to air pollution according to the definition of the proximity to the high-traffic roads within a buffer of 150 m and less exposed to “positive” environmental exposure by definition the proportion of green space or relation to the recommendations the meter square of green space per habitant by census blocks. This result was similar with previous studies in France (Lille, Marseille) which have reported that the most deprived neighborhood are located in census blocks with the highest level of NO_2_ concentrations [12], in Europe [60, 61], in Canada [62] or in United States [6]. Moreover, regarding residential surrounding greenness and proximity to green spaces, previous studies have reported that individual socioeconomic status could modify the health benefits of green spaces [63]. Dadvand et al in 2012, observed a larger benefit of green spaces for pregnant women with lower education qualifications [64, 65]. In this context, neighborhood socioeconomic status could also have a potential modifying effect on the association between green spaces and health.

We also demonstrated that the level of deprivation of the neighborhood have an impact on the infant and neonatal mortality risk. These finding are coherent with previous literature in France [66] and in other countries [67–69]. The explanations may be that during pregnancy, mothers are likely to face multiple stressful life events, including alone mother, unemployment, and little resources to deal with these socioeconomic conditions [70, 71] and parental factors include poor health status (for example, diabetes, obesity and chronic obstructive lung diseases), toxicants such as nicotine, caffeine, cocaine or alcohol [56, 58]. Previous article demonstrates that the employment status and individual education were strong predictors of spatial behavior related health services [72], individuals overcome spatial access barriers as the ability during pregnancy (individual mobility) and motivation (reasons to visit healthcare services) [73]. Moreover, literature has established that the neighborhood environment of mother and child has an influence on future birth outcomes independently of individual risk factors [74–76].

The major result we found is that despite a significant relation on infant and neonatal mortality risk according to the level of deprivation index, no environmental health inequalities has been revealed after taking into account the combined effect. An important feature of our study was that we assess environmental patterns of census bl0ocks according to the level of socioeconomic, the environmental level of exposure and the accessibility measure to healthcare diagnostics, which are very diverse definition by nature. While the deprived neighborhood in Nice are exposed to higher level of high-traffic roads and lower level of green space, they have higher density and shorter time/distance to gynecologist, obstetrics services, pediatrics and maternity. An English study shows that there is considerable variation in the distances from maternity depending on the geographical location [77], nevertheless population living in deprived neighborhood privileges shorter distance [72]. Centrality is a strong predictor of spatial behavior. Living in center was associated with less distance and time to facilities, an easy access and a higher offer of healthcare facilities for deprived neighborhood.

## Conclusion

Based on neighborhood deprivation, environmental exposure and accessibility to healthcare related to newborn and mother, we identified three environmental patterns at the census block level in Nice metropolitan area. We demonstrate that even if the most deprived census blocks are the most exposed to negative environmental exposure and have a higher risk of neonatal and infant mortality, they have higher availability, an easy accessibility of healthcare and very good transportation networks which allow us to not be exposed to environmental health inequalities. We focused on infant and neonatal mortality, important health indicators of the global health status of population. Quantitative measurements of inequality in geographic accessibility to pediatric care as well as that a mean distance or travel time is very important for priority setting to ensure fair access to facilities related to women and newborn. In this context, our analyses may be useful for influencing urban and public health policies aimed at promoting actions to census blocks identified with needs and their effects on health.

## Abbreviations

GIS, geographical information system; INSEE, the national institute of statistics and economic studies; IRIS, îlot regroupé pour l'information statistique; MA, metropolitan area;; MCA, multiple component analysis; NO_2_, dioxide azote; PM, particulate matter; WHO, World Health Organization
